# Toxins Targeting the K_V_1.3 Channel: Potential Immunomodulators for Autoimmune Diseases

**DOI:** 10.3390/toxins7051749

**Published:** 2015-05-19

**Authors:** Yipeng Zhao, Jie Huang, Xiaolu Yuan, Biwen Peng, Wanhong Liu, Song Han, Xiaohua He

**Affiliations:** 1 Department of Pathophysiology, School of Basic Medical Sciences, Wuhan University, Wuhan 430072, China; E-Mails: zhaoyipeng@whu.edu.cn (Y.Z.); jiehuang@whu.edu.cn (J.H.); yuanxiaolu@whu.edu.cn (X.Y.); 2 Hubei Provincial Key Laboratory of Developmentally Originated Disease, School of Basic Medical Sciences, Wuhan University, Wuhan 430072, China; E-Mail: pengbiwen@whu.edu.cn; 3 Hubei Province Key Laboratory of Allergy and Immunology, School of Basic Medical Sciences, Wuhan University, Wuhan 430072, China; E-Mail: liuwanhong@whu.edu.cn

**Keywords:** toxins, K_V_1.3 channel, effector memory T-cell, autoimmune diseases

## Abstract

Autoimmune diseases are usually accompanied by tissue injury caused by autoantigen-specific T-cells. K_V_1.3 channels participate in modulating calcium signaling to induce T-cell proliferation, immune activation and cytokine production. Effector memory T (T_EM_)-cells, which play major roles in many autoimmune diseases, are controlled by blocking K_V_1.3 channels on the membrane. Toxins derived from animal venoms have been found to selectively target a variety of ion channels, including K_V_1.3. By blocking the K_V_1.3 channel, these toxins are able to suppress the activation and proliferation of T_EM_ cells and may improve T_EM_ cell-mediated autoimmune diseases, such as multiple sclerosis and type I diabetes mellitus.

## 1. Introduction

Voltage-gated ion channels function as guards with regard to the ion permeability of cell membranes to regulate the intracellular concentrations of various ions, such as potassium (K^+^), sodium (Na^+^) and calcium (Ca^2+^). Because of selective evolution, venomous animals and plants have the ability to produce toxins targeted at these ion channels that cause paralysis, respiratory failure and death in their enemies. Peptide toxins from snakes, scorpions, spiders, sea anemones and cone snails have been widely used for structural and functional research addressing ion channels [1]. Therefore, there is a great potential for applying peptide toxins in designing drugs for ion channel diseases.

In the last few years, progress has been made in identifying the types of ion channels in lymphocytes and their roles in regulating Ca^2+^ signaling, activation, differentiation, secretion and migration.

The focus of potassium channels that regulate membrane potential in lymphocytes is the Shaker-related voltage-gated K^+^ channel K_V_1.3 (also known as KCNA3) and the Ca^2+^-activated K^+^ channel KCa3.1 (also known as IKCa1 or KCNN4). The expression levels of K_V_1.3 and KCa3.1 and their contributions to Ca^2+^ influx are different based on the types and activated state of T-cells. In autoimmune disorders, effector memory T (T_EM_)-cells encounter self-antigens and are activated, and the K_V_1.3 channels on the cell membrane increase significantly. Toxins targeting K_V_1.3 can selectively impact T_EM_ cell function, which suggests the regulatory role of K_V_1.3-blocking toxins in autoimmune diseases [2].

## 2. T-Cells and Autoimmune Diseases

An immune system distinguishes infectious pathogens from its own body components by a complex network. In general, most animals do not induce immunoreactions to autoantigens, forming self-tolerance. However, under certain conditions, an immune system will provoke positive immune responses to autoantigens. When the balance of immunoreaction and immunoregulation breaks down, autoimmune diseases may develop. Memory T-cells play a major role in the progression of these diseases.

Based on expression levels of chemokine CCR7 and phosphatase CD45RA, human memory cells can be sorted into central memory T (T_CM_) and T_EM_ cells. Both naive CD4^+^ and CD8^+^ cells express CCR7 and CD45RA and enter into lymph nodes with CCR7. In lymph nodes, naive T-cells are activated by antigens presented by antigen-presenting cells (APCs), release cytokines and differentiate into effector cells. When antigens are eliminated, most effector cells will undergo programmed cell death, but a few cells can survive and differentiate into long-lived T_CM_ cells. These cells also rely on CCR7 for entry into lymph nodes, but do not express CD45RA. When T_CM_ cells recognize the same antigens, they turn into effector cells and clear antigens more efficiently. In autoimmune diseases or chronic infections, antigens continually stimulate the immune system, and T_CM_ cells differentiate into T_EM_ cells. These cells live for a short time and develop into CCR7^−^CD45RA^−^ (exception: CD8^+^CCR7^−^CD45RA^+^T_EMRA_). They do not need to enter into lymph nodes and remain in the periphery to be ready for stimulation. When an antigen is encountered, T_EM_ cells differentiate into effector T-cells and migrate to an inflammatory site. Then, they release pro-inflammatory factors for a quick immune response. CD8^+^ T_EM_ cells also serve as cytotoxic T lymphocytes and release cytotoxicity factors, such as perforin and granzyme [3,4,5].

When a thymus matures, most autoreactive T-cells are eliminated, but a few cells escape to peripheral immune organs. Under normal conditions, these cells are suppressed by regulatory T-cells. In autoimmune diseases, this suppression mechanism disappears, and autoreactive T-cells attack target organs, causing tissue injury.

For example, multiple sclerosis (MS) is a typical autoimmune disease in which autoreactive T-cells migrate from peripheral blood into the CNS and attack axons, myelin sheaths and oligodendrocytes. This damage leads to demyelination and impacts neural signal transmission, which causes CNS dysfunction. The key event is when inflammatory T-cells permeate the blood brain barrier (BBB), invade CNS and are re-stimulated by autoantigens myelin basic protein (MBP), myelin oligodendrocyte glycoprotein (MOG) or myelin oligodendrocyte glycoprotein [6,7].

## 3. K_V_1.3 and Immune System Regulation

### 3.1. K_V_1.3 and KCa3.1 in the Immune System

Potassium channels are tetrameric membrane proteins that mediate K^+^ efflux to hyperpolarize the cellular membrane. Made up of 78 family members, the K^+^ channel family is the third largest family of signaling molecules in the human genome, following G-protein coupled receptors and protein kinases [8]. They are expressed on cells from both innate and adaptive immune systems.

The innate immune system includes natural killer (NK) cells, mast cells, eosinophils, basophils, and the phagocytic cells monocytes/macrophages, neutrophils and dendritic cells (DCs). These cells recognize “invaders” and release toxic molecules to non-specifically kill them. Moreover, macrophages and DCs, which present antigens to T-cells and play roles in adaptive immunity, are called antigen-presenting cells. Based on the published literature, most innate immune cells express K^+^ channels, but their functions in different cell types require further research.

In contrast, K^+^ channel expression has been systematically elucidated in the adaptive immune system. Patch clamping makes recording electrical signals from a single lymphocyte possible. K_V_1.3 and KCa3.1 expression levels are detected in the membranes of T-cells. The expression levels of these two potassium channels track the activation and differentiation of T-cells [9,10,11]. However, there are some differences in their mediation of the efflux of K^+^.

K_V_1.3 consists of four α-subunits arranged around a central pore as a homotetramer. Each subunit is composed of six transmembrane segments S1–S6 and a pore loop between S5 and S6. When membrane depolarization occurs, the arginine residues present in the S4 segment act as voltage sensors and cause a structure change and channel opening. In addition to T-cells, the K_V_1.3 channel is also expressed in other immune cells, such as B-cells [12] and macrophages [13].

KCa3.1, expressed in lymphoid organs, is a calcium-dependent K^+^ channel, the *C*-terminus of which binds to calmodulin. When Ca^2+^ interacts with calmodulin, the channel opens and strongly hyperpolarizes the membrane, which provides a force for Ca^2+^ entry [2,14].

**Figure 1:**
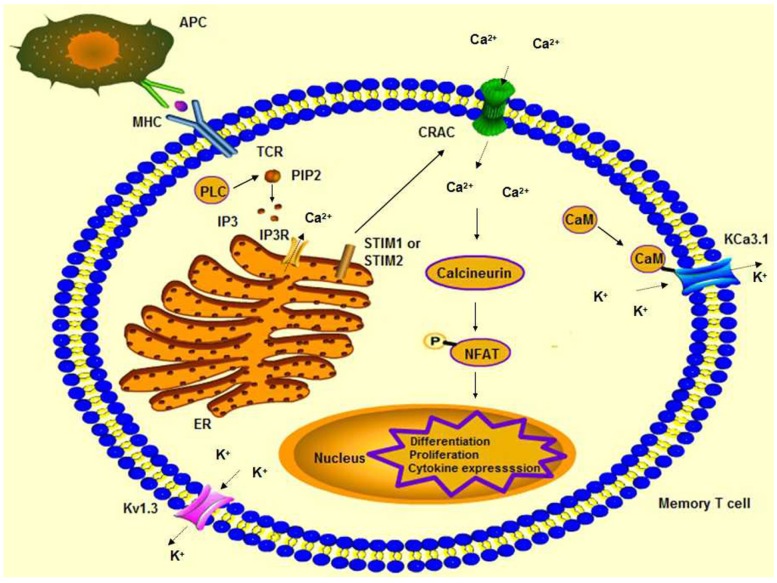
The influx of Ca^2+^ and efflux of K^+^ in the stimulation of memory T-cell. After, APC presents antigen to a T-cell, and IP_3_ is produced, which liberates Ca^2+^ from the ER. Meanwhile, CRAC channels are open and lead a Ca^2+^ influx. The increase of Ca^2+^ promotes the efflux of K^+^.

Each of these K^+^ channels regulate the membrane potential of T-cells and assist calcium release-activated calcium (CRAC) channels in promoting calcium influx, which is essential for T-cell activation. When an immune response occurs, an APC presents an antigen to a TCR. Then, IP_3_ is produced and binds to the IP_3_R, which releases Ca^2+^ from the endoplasmic reticulum (ER). Meanwhile, stromal interaction molecule 1 (STIM1) or STIM2 activates the opening of CRAC channels for Ca^2+^ entry. The Ca^2+^ influx results in gene expression through the calcineurin-NFAT pathway. The opening of K_V_1.3 and KCa3.1 induces the efflux of K^+^ to counterbalance the increase of Ca^2+^ in the cytoplasm [8,15] (Figure 1).

**Figure 2:**
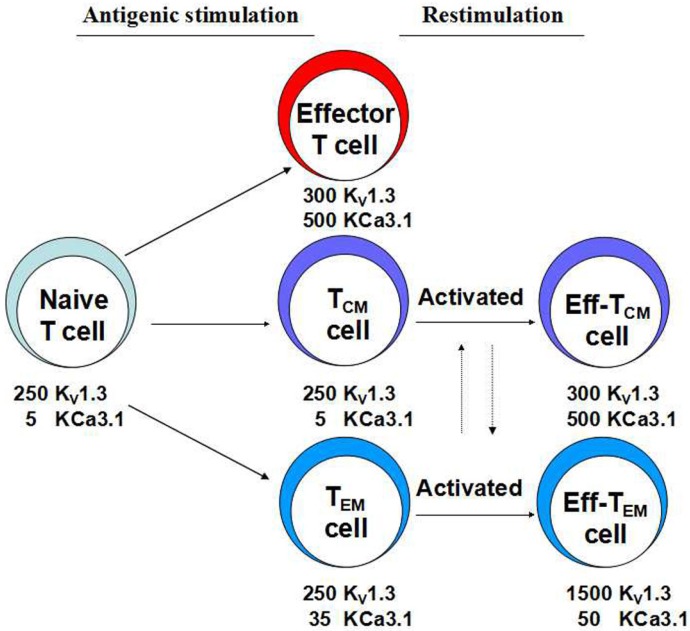
Expressions of K_V_1.3 and KCa3.1 channels in human T-cells. The expressions of both channels vary based on states of activation and differentiation. (Eff-T cell is effector T cell for short.)

The contribution of K_V_1.3 or KCa3.1 to the intracellular Ca^2+^ concentration is determined by their expression levels and varies in different subsets of T-cells. When human T-cells are inactive, they express 200–300 K_V_1.3 and 5–20 KCa3.1 per cell [16,17]. Following stimulation by antigens or mitogens, channel expression differences between effector T-cells arise. In naive or T_CM_ cells, the transcription and translation of KCa3.1 are significantly upregulated, and the number on the membrane can be increased to the levels of K_V_1.3 (KCa3.1 200–500, K_V_1.3 400–500 per cell) [16]. However, while K_V_1.3 expression increases (1,500 per cell), KCa3.1 levels stay nearly constant in T_EM_ cells [17] (Figure 2). This causes T_EM_ cells to produce various cytokines, including IL-2, IFN-γ, IL-4 and IL-17. These variances in expression affect membrane potential and calcium signaling, which regulate the expression of the downstream gene for T-cell activation and immune responses. As a result, targeting the K_V_1.3 channel inhibits T_EM_ cells activated by autoantigens in inflammation sites and is a promising strategy in remitting autoimmune diseases.

### 3.2. K_V_1.3 Channel Inhibitors

K^+^ channels mediate the efflux of K^+^ to hyperpolarize the plasma membrane when the membrane potential is depolarized. The α-subunits of K^+^ channels gather to form a heterotetramer relatively freely and are responsible for the different biophysical and pharmacological properties of cells. The β-subunits can interact with and modify the α-submit complexes [18]. K^+^ channel proteins can also be post-translationally modified by mechanisms, such as phosphorylation, ubiquitinylation and palmitoylation.

The diversity of the structural characteristics of the K^+^ channel family causes great challenges in drug discovery. However, this diversity is also promising for the highly selective targeting of specific types of homotetramers or heterotetramers.

There are three strategies for K^+^ channel regulator design: metal ions, organic small molecules and venom-derived peptides. The mechanism generally targeted is blocking the central pore of K^+^ channels to prevent K^+^ transport or modifying the structure of the voltage-sensor domain. For metal ions, such as Ba^2+^, Co^2+^ and Ni^2+^, the effect on K^+^ channel blocking is small and not selective. Thus, there is less focus on the metal ion strategy. The other two types of drugs can be used to treat immune system and nervous system diseases. In addition, toxins have advantages over small molecules due to their affinity and selectivity for specific channels. Toxins and their analogs can inhibit one type of potassium channels at the picomole level and display 1,000-fold selectivity for it over other potassium channels. The extremely high affinity and selectivity of toxins are difficult to achieve with chemically-designed molecules.

Venomous animals, such as snakes, scorpions, spiders, sea anemones and cone snails, can produce a variety of toxins that act on ion channels. The first toxin reported in the literature was the bee neurotoxin apamin, which blocks the KCa2 (SK) channel [19,20]. Subsequently, the scorpion toxins noxiustoxin (NTX) [21] and charybdotoxin (ChTX) [22] were discovered. NTX blocks K_V_1.2 (IC_50_ ≈ 2 nM) and K_V_1.3 (IC_50_ ≈ 1 nM), two members of the K_V_1 family. While the selectivity of ChTX is poor, it inhibits KCa1.1 in nM concentrations [22], as well as K_V_1.2 (IC_50_ ≈ 14 nM), K_V_1.3 (IC_50_ ≈ 2.6 nM) and K_V_1.6 (IC_50_ ≈ 22 nM) [23]. These toxins are applied in neuroscience to increase the physiological properties of K^+^ channels. However, their potential can be further exploited. These peptides may help us better study the structure of channel proteins, the composition of the subunits and the mechanism underlying K^+^ gating.

In the past 30 years, hundreds of toxin peptides targeting K^+^ channels have been discovered. The toxins contain between 18 and 60 amino acid residues and are assembled with 2–4 disulfide bridges, which makes them resistant to denaturation [24]. These peptides and their synthetic derivatives target K_V_ and KCa channels efficiently and selectively. However, Kir- and K2P channel-blocking toxins are rarely reported [25,26].

**Figure 3:**
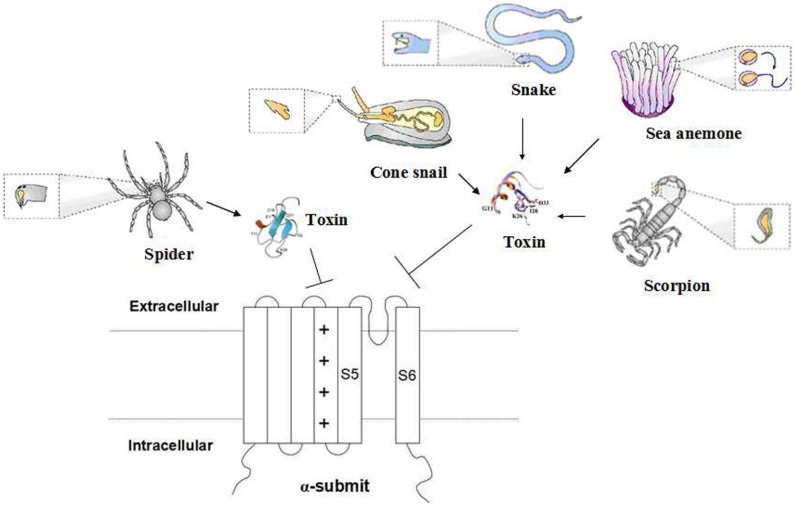
The mechanism of animal toxins blocking the potassium channel. Potassium channels function as a heterotetramer. Most toxins from animal venoms block the central pore to prevent K^+^ transport, while spider toxins regulate the voltage sensors.

The peptides modify K^+^ channels through two mechanisms. Toxins from scorpions, sea anemones, snakes and cone snails bind the outer vestibule of the channels and close the central pore by inserting a lysine side chain into it, similar to plugging a cork into a wine bottle; subsequently, peptides interact with the outside channel amino acids to stabilize their combination [8,27,28,29] (Figure 3). Spider toxins act on the voltage sensor domain of K^+^ channels, making the channel more difficult to open and increasing the possibility of a closed state [30,31]. As modified gating toxins, spider toxins bind to the voltage sensor domain and insert one side of a cluster of hydrophobic residues into the membrane to strengthen their bindings, but the insertion has no specificity, making drug design difficult (Figure 3).

Our goal is to search for K_V_1.3 blockers with potential as immunomodulators in autoimmune diseases. The first compound used for K_V_1.3 function research was 4-aminopyridine (4-AP). Although it blocks K_V_1.3 well and has been used in the clinic to treat MS, its poor selectivity leads to side effects, such as headaches and seizures [32]. Low selectivity is a common limitation of small molecules for the intended target. Submicromolar inhibitors of K_V_1.3 are also being explored and synthesized. The therapeutic blockers with the most potential focus on toxin peptides from scorpions and sea anemones because of their high affinities and selectivities.

Toxins derived from scorpion venom, sea anemone, snakes and other animals were found to block K_V_1.3 with different affinities and selectivities. Among these toxins, there are numerous scorpion toxins targeting K_V_1.3, including noxiustoxin (NTX), charybdotoxin (ChTX), margatoxin (MgTX), Orthochirus scrobiculosus toxin 1 (OSK1), kaliotoxin, agitoxin-2, hongotoxin and anuroctoxin [8]. As research progresses, more K_V_1.3-blocking toxins from various animals are being discovered that block K_V_1.3 with various IC_50_ values (Table 1).

**Table 1:** Toxins blocking the K_V_1.3 channel. The blocking potencies (K_d_ or IC_50_ values) of wild-type toxins and selected analogs on K_V_1.3 channels are shown. For comparison, K_V_1.1 and K_V_1.2 channels are included for selectivity.

Toxins WT/Analog	Derived from	K_V_1.3 K_d_-IC_50_ (nM)	K_V_1.1 K_d_-IC_50_ (nM)	K_V_1.2 K_d_-IC_50_ (nM)	Reference
Noxiustoxin	Scorpion	1	>25	2	[33]
Charybdotoxin	Scorpion	2.6	>1,000	14	[33]
Margatoxin	Scorpion	0.11	0.52	10	[34]
Kaliotoxin	Scorpion	0.41	1.1	25	[35]
Agitoxin-2	Scorpion	0.004	0.044	ND	[36]
Hongotoxin	Scorpion	86	31	170	[37]
Anuroctoxin	Scorpion	0.73	>100	6.1	[38]
OSK1	Scorpion	0.003	0.4	2.96	[39]
Vm24	Scorpion	0.029	35	7.5	[40]
ADWX-1	Scorpion	0.002	0.65	>100	[41]
ImKTX88	Scorpion	0.091	389	8,470	[42]
MTX-HsTX1	Scorpion	4	1	>100	[43]
ShK-Dap^22^	Sea anemone	0.023	1.8	39	[44]
ShK-186	Sea anemone	0.069	7	48	[44]
ShK-192	Sea anemone	0.14	22	>100	[44]
Gambierol	Dinoflagellate	853.5	64.2	34.5	[45]
ViTX	Cone snail	2,090	1,590	ND	[46]
AcK1	Worm	266	>1,500	ND	[47]
BmK1	Worm	6,900	ND	ND	[47]
BF9	Snake	120	ND	ND	[48]
Aurelin	Jellyfish	4,120	ND	ND	[47]

Abbreviation: OSK1, *Orthochirus scrobiculosus* toxin 1; Vm24, venom toxin 24 from *Vaejovis mexicanus smithi*; ADWX-1, autoimmune drug 1 from Wenxin group; ImKTX88, *Isometrus maculates* potassium channel toxin 88; MTX, maurotoxin; HsTX1, Heterometrus spinnifer (Scorpionidae) toxin 1; ShK, *Stichodactyla helianthus* toxin; Dap, Diaminopropionic acid; ViTX, *Conus virgo* toxin; AcK1, *Ancylostoma caninum* potassium channel toxin 1; BmK1, *Brugia malayi* potassium channel toxin 1; BF9, *Bungarus fasciatus* toxin 9; ND, not detected.

Although the majority of peptides can block K_V_1.3 in the nanomolar to picomolar range, their selectivities for the channel are not satisfactory. In order to achieve better results, chemical modifications and point mutation techniques are being applied to the original toxin molecules. The first analysis of these molecules will investigate which residues of the toxins should be modified for higher selectivity binding.

For example, ShK, a peptide from the Caribbean sea anemone, was found to have a high affinity for K_V_1.3 (K_d_ ≈ 11 pM) [49]. Additionally, it blocks the K_V_1.1, K_V_1.4 and K_V_1.6 channels at picomolar concentrations. Subsequently, using a thermodynamic mutant cycle analysis to study residues in toxin binding to the mK_V_1.3 channel, ShK-Dap^22^ was designed. A non-natural amino acid known as diaminopropionic acid was substituted for lysine^22^, which is known to be critical for the binding of many toxins. Although this mutant has a lower affinity for mK_V_1.3 (K_d_ ≈ 23 pM) compared to ShK, its selectivity over the other K_V_1.x channels and IKCa1 was remarkably improved [50]. On this basis, a further study revealed the different mechanisms controlling the binding of these two toxins to mK_V_1.3. The wild-type toxin inserts Lys^22^ into the central pore and interacts with residue Tyr^400^; this strong interaction was replaced by Dap^22^ and His^402^ in the ShK-Dap^22^ binding. Because of the weak affinity of the interaction between Dap^22^ and the residues of other channels (*i.e.*, K_V_1.1, K_V_1.4, K_V_1.6) compared to Lys^22^, ShK-Dap^22^ has a high selectivity for K_V_1.3 [51]. This reveals that minute differences in amino acids may lead to pronounced changes in dynamics. Besides affinity and selectivity, stability also needs to be taken into account. ShK-186 retains the selectivity and potency profile of ShK analogs and minimizes digestion by carboxypeptidases by replacing the C-terminal carboxyl with an amide [44].

In addition to small-scale changes in residues, toxins can be combined with other regions for special purposes; this requires analyzing the binding domain affinity that can be used for multi-target, high-selectivity drug design. Maurotoxin (MTX) is an effective SK channels blocker, and HsTX1 inhibits K_V_1.1 and K_V_1.3. MTX-HsTx1 contains the N-terminal helical region of MTX and the C-terminal β-sheet region of HsTx1 for binding SK channels, mK_V_1.1 and mK_V_1.3 [43].

## 4. K_V_1.3 Blocking Toxins for the Therapy of Autoimmune Diseases

In general, autoimmune diseases involve the stimulation of T-cells at a peripheral region and a target site. The occurrence and progression of these processes are difficult to pinpoint clinically because of tracking, observation and sample collection difficulties. Animal models may help us to better understand these diseases and aid in future drug designs.

However, to test K_V_1.3 blocking, the practicability of mice models is limited. T-cells of mice express fewer and different potassium channels [52,53]. When stimulated, the numbers of K_V_1.3 and KCa3.1 both increase in T_EM_ cells (K_V_1.3 from 300–800 and KCa3.1 from 130–400) [5], which indicates that the application of K_V_1.3 blockers in a mouse model is not convincing for effective T_EM_ targeting.

Rat autoimmune disease models, by contrast, are more persuasive. Although the expression of potassium channels on T-cell membranes is also different from that of humans in a quiescent state, after stimulation, the rat T_EM_ phenotype upregulates K_V_1.3, and the T_CM_ phenotype maintains K_V_1.3 at low levels. This difference is similar to that in humans, which suggests that rat models are better for evaluating the role of T_EM_ cells in T-cell-mediated autoimmune diseases and the therapeutic effects of K_V_1.3 blockers [49,54].

### 4.1. Multiple Sclerosis

Multiple sclerosis (MS) is a chronic inflammatory disease characterized by myelin damage in the central nervous system (CNS). The clinical symptoms of this disease are decreased vision, eye pains, dysphagia, limb weakness and difficulties in walking. There are millions of patients around the world, the majority of whom are women. The incidence of MS continues to increase. A small number of patients can be cured after the first attack, but most patients have relapsing and remitting disease [7].

Clinical samples from MS patients (brain, spinal cord and cerebrospinal fluid) are not easy to get, which cannot satisfy the research for potential drugs. Therefore, animal models have been generated to study MS. These models fall into three categories: gene mutation or chemically (*i.e.*, cuprizone)-induced white matter lesions for myelination and remyelination research [55], virus-induced models for etiology studies and autoimmune cell-mediated immune responses for neuroinflammation studies. The classic model is experimental autoimmune encephalomyelitis (EAE), in which the imbalance of immune activation and immunoregulation leads to worsening CNS injury; this model has the most similar pathogenesis to MS and is generally used for drug screening [56,57,58].

In the 1980s, it was reported that after being stung by a scorpion, an MS patient had reduced disease [59]. This observation suggests that peptides from scorpion venom may improve MS. As a typical autoimmune disease, MS and its animal models are regularly studied in research into K_V_1.3-blocking toxins.

In the Sprague-Dawley (SD) rat acute EAE model, ADWX-1, an analog of scorpion toxin BmKTX [41], has been shown to exhibit preferential, dose-dependent inhibition of CD4^+^CCR7^−^ T_EM_ cells, which are perpetrators of EAE. ADWX-1 reduces the number of activated Th1 and Th17 cells and also decreases the secretion of inflammatory factors IL-2, IFN-γ and IL-17. In contrast, it has no effect on regulatory Foxp3^+^ cells, which are the antagonists of autoreactive cells [6].

ShK-186 [60], which was tested in the Dark Agouti (DA) rat EAE model (an animal model that is more similar to chronic relapsing-remitting multiple sclerosis), can also decrease inflammatory T-cell infiltration into the CNS and the demyelination process [60]. This toxin peptide has been used in clinical trials.

It is important to emphasize the selectivity for K_V_1.3 in EAE models. Based on the electrophysiological evidence, the K_V_1.1 and K_V_1.2 of myelinated axons are located under the myelin sheath [61,62]. Toxin peptides, the nanomolar molecules associated with K_V_1.x, can easily enter into the CNS after the BBB has been broken down by inflammatory cells. These peptides may then influence the normal physiological functions of neurons. A goal in this field is to obtain good clinical results without neurological impairment.

### 4.2. Rheumatoid Arthritis

Rheumatoid arthritis (RA) is a systemic autoimmune disease with widespread concerns. The joint changes are mediated by the dysregulated and invasive growth of the synovial cells developing into what is referred to as a pannus that overlays and destroys cartilage and bone. CCR7^−^K_V_1.3^high^ T_EM_ cells have been identified from synovial fluid of RA patients, suggesting that K_V_1.3 blockers may function as immunomodulators in RA [63].

A chronic arthritis model is DA rats injected with pristane oil, which induces RA [64]. Disease severity in this model worsens with time. The immune regulation of drugs can be evaluated by measuring the degree of ankle welling and detecting inflammatory factors (*i.e.*, TNF-α) in synovial fluid. Subcutaneous injections of ShK-186 at the onset of disease signs can ameliorate the severity of RA, including reducing inflammatory infiltration and tissue damage [63]. ShK-186 has been found to preferentially suppress T_EM_ cells from synovial fluid of RA patients [63]. However, there is no difference when the K_V_1.3 blocker margatoxin (MgTX) is applied to peripheral lymphocytes from patients with rheumatoid arthritis or healthy individuals. This is because most T-cells in circulation remain in a less-activated state [65].

### 4.3. Type I Diabetes Mellitus

Type I diabetes mellitus (TIDM) is caused by a loss of beta cells due to autoimmune-mediated destruction. The target antigens recognized by autoimmune cells include insulin, glutamate decarboxylase (GAD) and membrane proteins of islet beta cells. Severe inflammatory infiltration in the islets of patients with HLA-II molecules expressed on beta cell membranes is characteristic of TIDM.

Biobreeding/Worcester (BB/Wor) rats develop experimental autoimmune diabetes mellitus after 70 days. The incidence can reach 100% in 110 days. 5-(4-phenoxybutoxy)psoralen (PAP-1) is a selective small molecule inhibitor of K_V_1.3 [66]. PAP-1 administration decreases the clinical manifestation in diseased rats. The rats have less T-cell and macrophage infiltration and less beta cell destruction [63]. Studies have reported that potassium channels are involved in glucose-stimulated insulin secretion in pancreatic beta cells, and K_V_1.3 specifically mediates insulin sensitivity in peripheral target tissues. No increase in basal or insulin-stimulated glucose uptake or adiponectin secretion has been reported in PAP-1-treated rat adipocytes, suggesting the effect of PAP-1 on diseased rats works through immunoregulation [67].

ShK-186 delays the onset of diabetes, but has no influence on disease incidence. This may due to the different pharmacokinetic properties of ShK-186, which has a net basic charge that makes concentrating in the pancreas difficult [44,63].

Lymphocytes from peripheral blood of TIDM patients are more sensitive to K_V_1.3 inhibition than healthy individuals, probably because of the enhanced K_V_1.3 expression in TIDM patients. An increase of T_CM_ cells has also been detected, suggesting that TIDM lymphocytes remain in a pre-activated state in these patients. However, MgTX also fails in modulating TIDM lymphocytes compared with healthy individuals. This indicates that autoantigen-specific T-cells require further investigation [68].

### 4.4. Rapidly Progressive Glomerulonephritis

Glomerulonephritis (GN) is usually initiated by infections or self-antigens. Immunostimulatory pathogen-associated molecular patterns (PAMPs) or damage-associated molecular patterns (DAMPs) activate both innate and adaptive immune systems. APCs promote the stimulation and differentiation of CD4 helper cells. CD4 Th1 cells mediate tissue damage through macrophages and basophils, while Th17 cells damage glomeruli directly [69].

In rats with anti-glomerular basement membrane glomerulonephritis (anti-GBM GN), K_V_1.3 expression was detected in glomeruli and some tubules. A flow cytometry analysis of kidney cells and immunofluorescence staining of lymphocytes revealed that these cells were CD4^+^ and that a few were CD8^+^ K_V_1.3^high^ T_EM_ cells. Moreover, rats injected with the small-molecule K_V_1.3 blocker Psora-4 showed less proteinuria and fewer crescentic glomeruli [70], suggesting the potential role of K_V_1.3 toxin peptides in the treatment of autoimmune glomerulonephritis.

### 4.5. Psoriasis

Psoriasis, one of the common inflammatory disorders of the skin, affects approximately 2.0%–2.5% of the world’s population. The most common symptom of psoriasis is patches of thick red skin often covered with silvery scales [71]. Psoriasis plaques and synovial fluid from psoriasis/psoriatic arthritis (PsA) patients exhibit significantly increased K_V_1.3^+^ memory T-cells compared to non-lesion psoriatic skin, normal skin or peripheral blood lymphocytes. Moreover, treatment with the K_V_1.3 blocker PAP-1 inhibits T-cell proliferation in the skin and joints of psoriasis patients and the secretion of cytokines IL-2 and IFN-γ [72].

Transplanted psoriatic plaque skin samples from psoriasis patients onto severe combined immunodeficient (SCID) mice results in the development of psoriasis lesions similarly to patients. When grafted mice are treated with ShK, a significant decrease in K_V_1.3^+^ cells is observed along with a marked therapeutic effect, including a normal appearance of the skin grafts [73]. In addition, PAP-1 can also alleviate psoriasis by reducing the hyperkeratosis, acanthosis and immune cell dermal infiltrates in the plaques [72].

## 5. Conclusions

K_V_1.3 channels mainly regulate calcium signaling during the activation of CCR7^−^ T_EM_ cells and maintain membrane potential by counteracting the influx of Ca^2+^ with the efflux of K^+^. Because T_EM_ cells play the major role in tissue injury, mediating chronic autoimmune response in autoimmune diseases by blocking K_V_1.3 is an effective way to treat these disorders. Based on this strategy, toxins targeting K_V_1.3 have been tested in various animal models of autoimmune diseases and samples from patients.

Although there are numerous toxins targeting K_V_1.3, because of poor affinity or selectivity, some of them only have applicability as tools for K_V_1.3 characterization research in autoimmune diseases. While numerous electrophysiology studies of new synthetic K_V_1.3-blocking toxin peptides are being reported, further *in vivo* experiments are required. ShK-186, an analog derived from ShK, has been used in clinical trials, suggesting the possibilities for autoimmune disease treatment. Toxin peptide researchers should further study toxins with higher affinity and selectivity through investigations using animal models.
